# P18/Stathmin1 is regulated by miR-31 in ovarian cancer in response to taxane

**DOI:** 10.18632/oncoscience.143

**Published:** 2015-03-23

**Authors:** Mohamed Kamel Hassan, Hidemichi Watari, Takashi Mitamura, Zainab Mohamed, Sherif F. EL-khamisy, Yusuke Ohba, Noriaki Sakuragi

**Affiliations:** ^1^ Department of Obstetrics and Gynecology, Hokkaido University Graduate School of Medicine, Sapporo, JAPAN; ^2^ Bitechnology Program, Zoology Department, Faculty of Science, Port Said University, Port Said, EGYPT; ^3^ Center of Genomics, Hemly Institute for Medical Sciences, Zewail City for Science and Technology, Giza, EGYPT; ^4^ Krebs Institute, Department of Molecular Biology and Biotechnology, University of Sheffield, UK; ^5^ Department of Cell Physiology, Hokkaido University Graduate School of Medicine, Sapporo, JAPAN

**Keywords:** ovarian cancer, chemoresistance, miR-31, taxane, Stathmin 1

## Abstract

MicroRNAs (miRNAs) have been reported to regulate the development of chemoresistance in many tumors. Stathmin 1 (STMN1) is a microtubule-depolymerizing molecule, involved in chemo-response; however, the mechanism of its regulation is unknown. Herein, the immunohistochemical study indicated significant upregulation of the STMN1 in the ovarian cancer tissues defined as resistant tumors compared with those defined as responsive tumors. STMN1 level elevated in the chemoresistant ovarian cancer cells, KF-TX, compared with the parental, KF, ones. Targeting STMN1 by siRNA restored taxane-sensitivity of KF-TX cells. Screening miRNA profiles from KF/KF-TX cellular set followed by bioinformatics-based prediction, revealed that miR-31 could be a possible regulator of STMN1. Down-modulation of miR-31 was verified by quantitative RT-PCR in the cellular set used. Overexpression of miR-31 in KF-TX cells (KF-TX-miR-31) significantly restored chemo-response and reduced STMN1 expression as well. STMN1 reduction-associated cellular characteristics such as enhanced microtubule polymerization and stability, as indicated by acetylated tubulin quantification, confocal visualization, and G2 phase delay, were observed in KF-TX-miR-31 cells, indicating the functional reduction of STMN1. miR-31 suppressed the luciferase activity in reporter construct containing the STMN1 3′-untranslated region (3′-UTR), confirming that miR-31 directly targets STMN1. miR-31 has therapeutic potency when introduced into ovarian cancer, in combination with taxane.

## INTRODUCTION

Epithelial ovarian cancer is the most frequent cause of gynecologic malignancy-related mortality in women [1, 2]. A complete clinical response can be achieved in approximately 80-90% of patients with early-stage disease and in 50% of patients with advanced-stage disease. The main limitation to a successful treatment is the development of resistance to combined chemotherapy, platinums coupled with taxane (TX). Even after achieving clinical remission after completion of initial treatment, most patients with advanced epithelial ovarian cancer will ultimately develop recurrent disease [3,4,5]. Recently, mechanisms of resistance to TX and platinum have been widely studied in ovarian cancers, which include genetic/epigenetic alterations and apoptotic defects. However, until now identification of master regulator(s) governing several molecular mechanisms related to chemoresistance yet to be well identified. These regulators are located in our interest because they can act as targets to efficiently overcome chemoresistance.

TX was originally isolated from the bark of the yew tree, and belongs to the antimicrotubule chemotherapeutic agents. TX kills the cancer cells by binding the β-tubulin and stabilizing the microtubules (MTs), causing the MT to persist depolymerization and arresting the cells in the G2/M phase followed by apoptosis. Thus, MT dynamic is involved in the response to TX probably by regulating factors that keep the MT stabilization/destabilization balance [6, 7]

Stathmin1 (STMN1, also known as oncoprotein 18 (Op18), p17, p18, p19, 19K, prosolin and metablastin) is a recently identified as tubulin-binding, cytosolic protein, which acts as a potent regulator of MT stability [8,9,10]. STMN1 sequesters soluble tubulin dimers and stimulates depolymerization at MT plus ends [11,12]. These two functions appear to reside in different regions of the protein [13]. Although the primary biological task of STMN1 appears to be controlling the MT structure, recent reports suggest it to have significant roles in human diseases and development of the nervous system [14-16]. STMN1 has been found to be overexpressed in neuroblastoma [17,18] and is upregulated in vincristine-resistant neuroblastoma cells [19]. STMN1 upregulation was also reported in the highly proliferative breast cancers and in ovarian cancers [20-22]. Stage-dependent expression of STMN1 has also been noted in ovarian cancers [23]. Depleting STMN1 in many cancer cell lines slows cell proliferation and ultimately leads to apoptosis [24-28]. Such apoptosis was proposed to be due to a delay during the G2 phase of the cell cycle [28].

MicroRNAs (miRNAs) are a class of 22-nucleotide noncoding RNAs, which are evolutionarily conserved and function as negative regulators of gene expression in a sequence-specific manner. miRNAs bind to complementary sequences in the 3′-untranslated region (UTR) of the target gene transcripts, leading to mRNA degradation and/or translational repression [29]. Aberrant levels of miRNA have been reported in a variety of human cancers, including ovarian cancer. [30-34]. Some miRNAs have been suggested to have both diagnostic and prognostic potencies while some others constitute novel targets for cancer treatment [35]. Recently, the role for miRNAs in determining drug sensitivity/resistance has emerged [36]. The current rapid advances in oligonucleotide/nanoparticle therapy create realistic optimism for the establishment of miRNAs as a new and potent therapeutic target and/or chemoresistant modulator in cancer treatment. On the other hand, the role of miRNA in the acquisition of drug resistance by ovarian cancer cells is still elusive.

Thus, we aimed to investigate the possible role of STMN1 in the chemoresponse in ovarian cancer and to study the regulatory mechanism of STMN1 expression under the effect of TX. We first confirmed the relationship between STMN1 expression and chemoresistance in ovarian tumor tissues. Then, functional studies were carried out to validate the regulatory mechanism of the expression of STMN1 by miR-31 in ovarian cancer cells and tissues.

## RESULTS

### STMN1 expression is elevated in chemoresistant ovarian cancer cells and tissues

**Figure 1:**
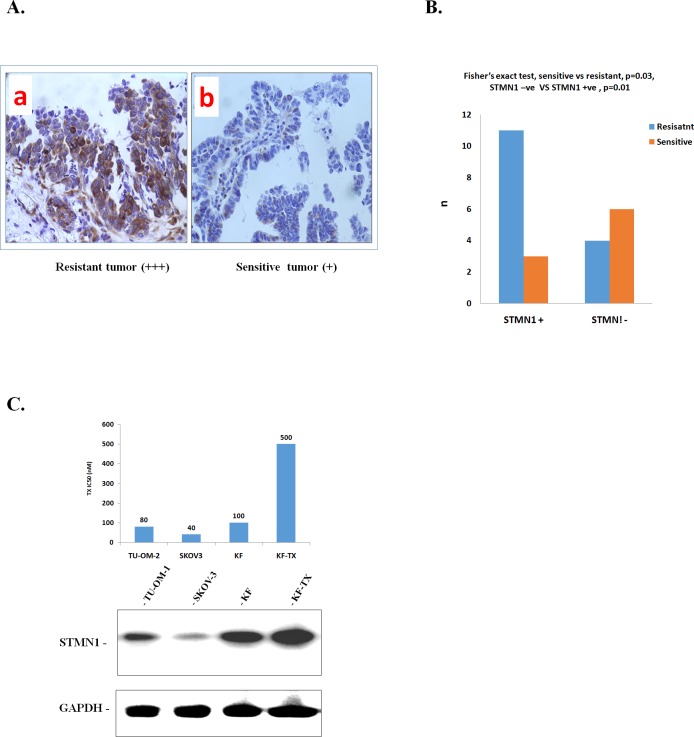
STMN1 expression increased with chemoresistance to TX in human ovarian cancers A. Representative results of IHC with high expression (+++; a) or very weak expression (+; b) are shown as photographs. B. Conclusive diagram shows the relationship between the expression of STMN1and chemoresponse In the ovarian cancer tissues examined. The high STMAN expression is significantly correlated with chemoresistance. From 24 ovarian cancer tissues samples, 14 tissue samples showed positive STMN1 expression. Of them, 11 samples (78.5%) were chemoresistant while 3 samples (21.5%) were chemoresponsive. On the other hand, ten samples showed negative or very week STMN1 expression. C. Western blotting analysis of STMN1 expression in four cell lines. Chemoresistant KF-TX cells express more STMN1 compared with parental KF cells. The blot shows that STMN1 expression is associated with IC50 to TX. SKOV-3, lowest IC50, shows the lowest expression level of STMN1.

To evaluate the possible relationship between STMN1 expression and development of ovarian cancer chemoresistance, we examined the expression level of STMN1 in surgical specimens from 24 human ovarian cancer tissues (Table.1). All cases were women with serous adenocarcinomas with FIGO Stage IIC, IIIC or IV [39], who underwent surgery as their initial treatment. Since the initial surgery was suboptimal in all patients, the subjects were stratified by response to subsequent taxane-containing chemotherapy (more than three courses in all cases) according to the Response Evaluation Criteria In Solid Tumors (RECIST) [40]. The 24 tumors were divided into two groups, sensitive and resistant, based on the response to chemotherapy within the first year of treatment (see materials and methods). In Table 1 the expression levels of STMN1 in the 24 tissue samples were summarized and most of the tissue samples with high STMN1 expression (represented in Figure.1A.a) were located in the chemoresistant category compared with the weak or negative expression (represented in Figure. 1A.b) tissues for STMN1. We found that, among the 24 tissue samples, 14 samples showed positive STMN1 expression. Most of them (11 samples; 78.5%) were categorized as chemoresistant tumors while 3 tumor tissues (21.5%) were categorized as sensitive tumors. On the other hand, 10 tissue samples showed negative or very weak STMN1 expression, 6 (60%) and 4 samples (40%) of which were categorized as chemosensitive and chemoresistant, respectively (Figure .1B).

**Table 1:** Differentia expression of STMN1 and miR-31 in ovarian cancer tissue samples

Case No.	Age	FIGO state	Histology	Chemo-response status	STMN expression +/−	STMN expression (intensity)	miR-31 expression (miR-31/RNU4)
1	51	IV	Serous	Resistant (SD)	+	+++	0.00
2	52	IV	Serous	Resistant (SD)	+	++	0.01
3	54	IIIV	Serous	Resistant (SD)	+	++	0.06
4	48	IV	Serous	Resistant (PD)	+	+++	0.06
5	48	IV	Serous	Resistant (SD)	+	++	0.07
6	38	IIIC	Serous	Resistant (PD)	−	−/+	0.24
7	49	IIIC	Serous	Resistant (PD)	+	+	0.40
8	67	IIIC	Serous	Resistant (PD)	+	+++	0.56
9	72	IV	Serous	Resistant (PD)	+	++	0.61
10	56	IIIC	Serous	Resistant (PD)	+	++	0.77
11	51	IV	Serous	Resistant (PD)	+	++	1.32
12	42	IIC	Serous	Resistant (PD)	−	−/+	1.50
13	53	IIIC	Serous	Resistant (PD)	−	−+	1.87
14	52	IIIC	Serous	Resistant (PD)	−	−/+	1.99
15	49	IIIc	Serous	Resistant (PD)	+	++	3.24
16	66	IV	Serous	Resistant (PD)	+	++	0.17
17	42	IIIC	Serous	Sensitive (PR)	−	−/+	0.50
18	47	IIIC	Serous	Sensitive (PR)	−	−	0.69
19	52	IV	Serous	Sensitive (PR)	+	+	1.00
20	47	IIIC	Serous	Sensitive (PR)	−	−	1.08
21	66	IIIC	Serous	Sensitive (CR)	−	−/+	2.14
22	51	IV	Serous	Sensitive (PR)	−	−	3.89
23	66	IV	Serous	Sensitive (PR)	+	++	7.02
24	47	IIIC	Serous	Sensitive (PR)	−	−/+	165.59

In order to investigate STMN1 regulation in response to TX *in vitro* in a subsequent study, we checked STMN1 expression in four ovarian cancer cell lines including TX-sensitive, parental KF cells and their TX-resistant counterparts, KF-TX cells. The expression level of STMN1 was associated with the TX IC_50_ for each cell line used. KF-TX cells (IC_50_: 500 nM) significantly expressed more STMN1 compared with parental KF cells (IC_50_: 100 nM) while SKOV-3 (IC_50_: 45 nM) showed the lowest STMN1 expression level (Figure 1C).

To verify the direct effect of TX on STMN1 expression, we treated both KF and KF-TX cells with TX at different doses (0nM to 200nM). STMN1 expression was directly affected by the increase in TX dose when treated for two days in both cells, however the maximum upregulation was also very high in KF-TX cells compared with KF cells which showed relatively limited upregulation even when treated with 200nM (double of IC_50_; supplementary data, S.1).

### SiRNA against STMN1 modulates sensitivity of KF-TX cells to TX

**Figure 2:**
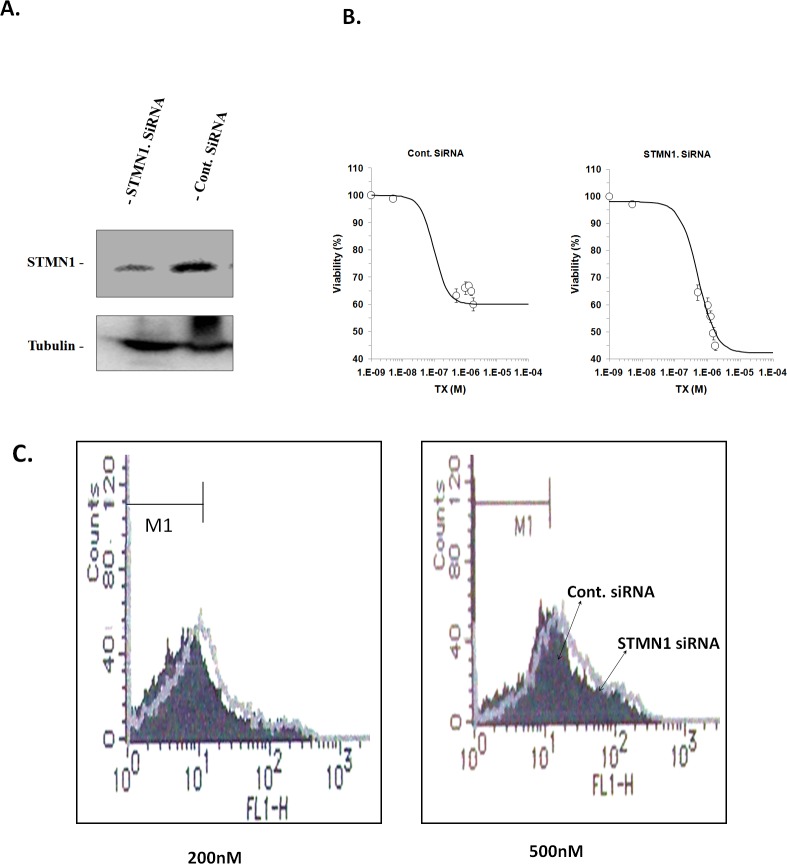
STMN1 knock down enhanced apoptotic cell death by TX in KF-TX cells A. A representative western blot shows the amendment of STMN1-specific but not control siRNA in KF-TX cells. Tubulin immunoblot was used as a loading control. The experiment was repeated three independent times for reproducibility. B. KF-TX cells were transfected with STMN1 siRNA or control siRNA (100 nM) twice. One day after last transfection, equal cell numbers were subcultured for further 24h, and then treated with TX for three days. The dose response curves are shown as Hill equations while each data point represents mean of three experiments; bars denote S.E;, C. Annexin V staining after TX in STMN1 KD cells compared with control. KF-TX were treated after SMN1 KD as well as in B. and then stained by annexin V and acquired by FACS analyzer. The cytograph shows a significant enhancement of TX-induced apoptosis as indicated by annexin stained cells.

To determine whether STMN1 protects ovarian cancer cells from TX-induced cell death, siRNA oligomers specific for STMN1 mRNA was used to knock down STMN1 expression. Transfection of STMN1 siRNA, but not control siRNA (Cont-siRNA), into KF-TX cells reduced expression of STMN1 (Figure 2A). To evaluate the benefits of targeting STMN1 in sensitizing ovarian cancer cells to TX, cellular viability at various doses of TX was studied in both STMN-siRNA and control-siRNA transfected KF-TX cells. Under these experimental conditions, Figure 2B and supplementary data (S.2) show significant reduction in cell viability of KF-TX, pre-treated with STMN1-siRNA, under different doses of TX than those pre-treated with control-siRNA then TX. Annexin V staining of KF-TX cells treated with TX (200 nM and 500 nM) with or without STMN1 knock down verified the enhanced cell death by TX in the STMN1-depleted KF-TX cells versus control (Figure 2C).

### Identification of putative STMN1-targeting miRNAs

**Figure 3:**
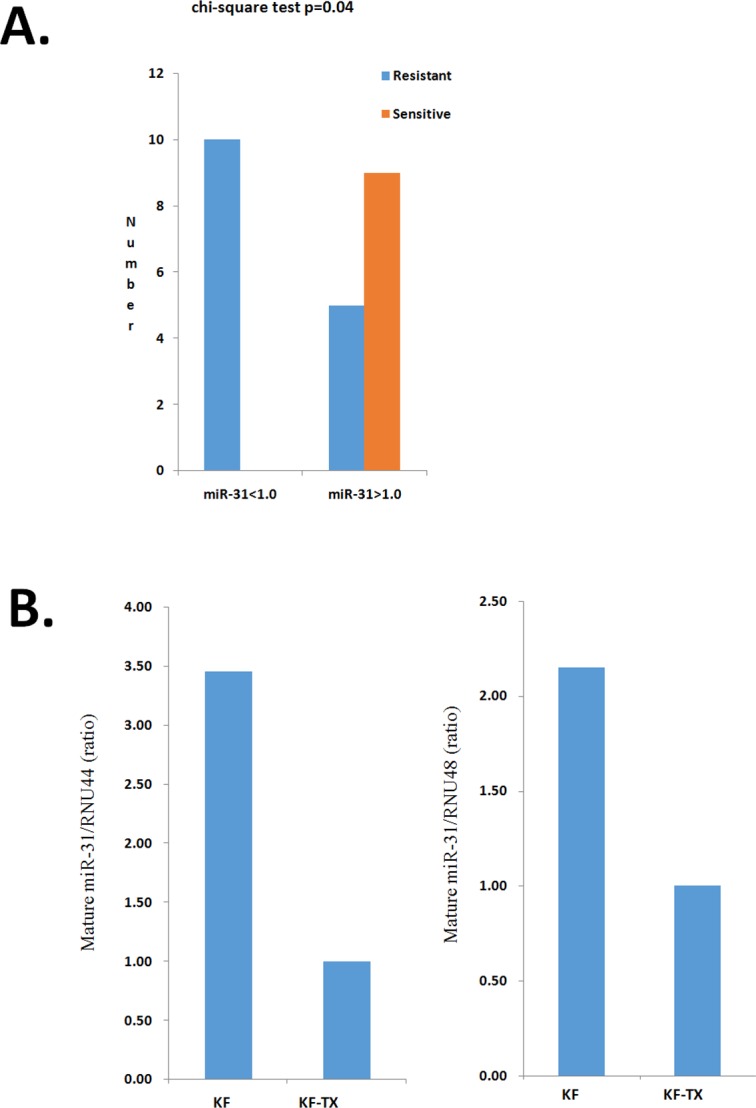
Differential expression of miR-31 in ovarian cancer tissues and cells **A.** Conclusive histogram shows the relationship between the expression of miR-31 and chemoresponse in the ovarian cancer tissues examined. The low miR-31 expression is significantly correlated with chemoresistance. From 24 ovarian cancer samples, 10 tissue samples showed relative low expression of miR-31 (ct < 1.0), all of them (100%) were chemoresistant while 14 tissue samples show high miR-31 expression (ct >1.0) 9 of them (64%) were categorized as chemosensitive and 5 samples (36%) were located in the chemoresistant category. B. Real-time PCR analysis show the expression levels of miR-31 in parental (KF) and TX-resistant (KF-TX) cells. *P <0.05.

Since aberrant STMN1 expression might contribute to chemoresistance acquisition of ovarian cancer cells, exploration of the mechanism responsible for STMN1 upregulation might be of great importance to overcome chemoresistance. Since miRNAs is emerging as key negative regulators of gene expression by mRNA degradation or translation inhibition [29], we thought that the differential expression profiles of miRNA might contribute to the aberrant expression pattern of STMN1. For that, we revisited the miRNA profile that we established previously in KF/KF-TX cellular set [41]. The bioinformatic-based prediction indicated that, among the STMN1 targeting miRNAs, we found miR-31 was significantly down modulated in KF-TX cells compared with KF cells by using two different miRNA target programs (TargetScan and miRBase). Since miR-31 was recently reported to be involved in the ovarian biology [42], we then re-examined the clinical tissue samples regarding its expression, and noticed that most of the tissue samples with low miR-31 expression (ct < 1.0) are categorized as chemoresistant tumors (10 samples; 100%). On the other hand, 14 tissue samples showed high miR-31 expression (ct >1.0), 9 of them were chemosensitive (64%) while 5 of them were chemoresistant (Figure 3A and Table 1). Importantly, the expression of miR-31 showed inverse relationship with STMN1 expression in the same set of samples with marginal significance (*P* = 0.07). We further studied the expression level of miR-31 in the KF/KF-TX cell set. The RT-PCR shows that miR-31 is significantly downregulated in KF-TX cells compared with KF parental cells (Figure 3B). These results concludes that miR-31 is inversely correlated to STMN1 expression, in the cellular set used, indicating a possible causal link between elevated STMN1 expression and miR-31 downregulation in chemoresistant ovarian cancer cells.

### Overexpression of miR-31 results in downregulation of STMN1 and restores chemosensitivity in KF-TX cells

**Figure 4:**
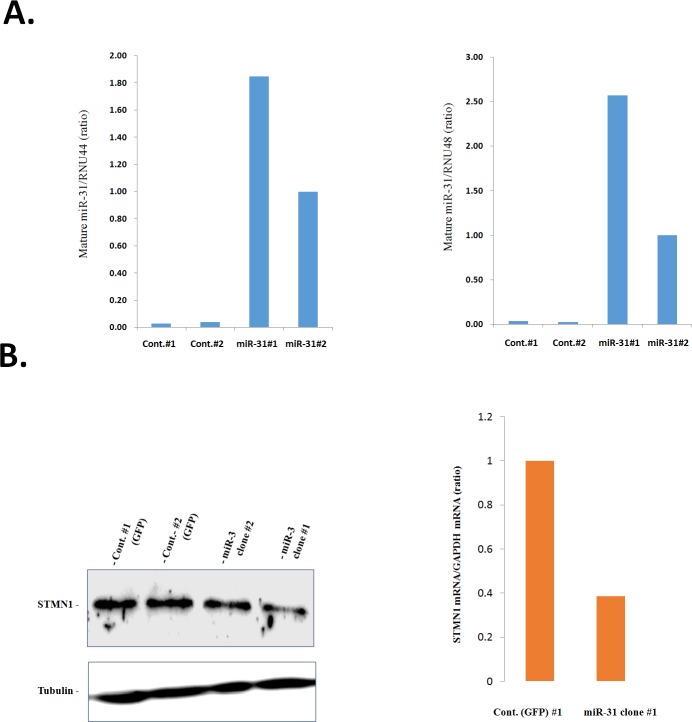
Establishment of miR-31 overexpressing clones **A.** Representative RT–PCR show the expression levels of miR-31 in cell clones named as clones (#1) and (#2) are high and middle, respectively B. STMN1 expression responses to miR-31 expression. Immunobloting analysis of STMN1 in the miR-31 overexpressing clones and control ones. The KF-TX-miR-31 show less expression level of STMN1 compared with control (left panel). Representative RT-PCR in one stable clone (#1) and its control shows that the STMN1 transcript level decreased compared with the control clone (right panel)

**Figure 5:**
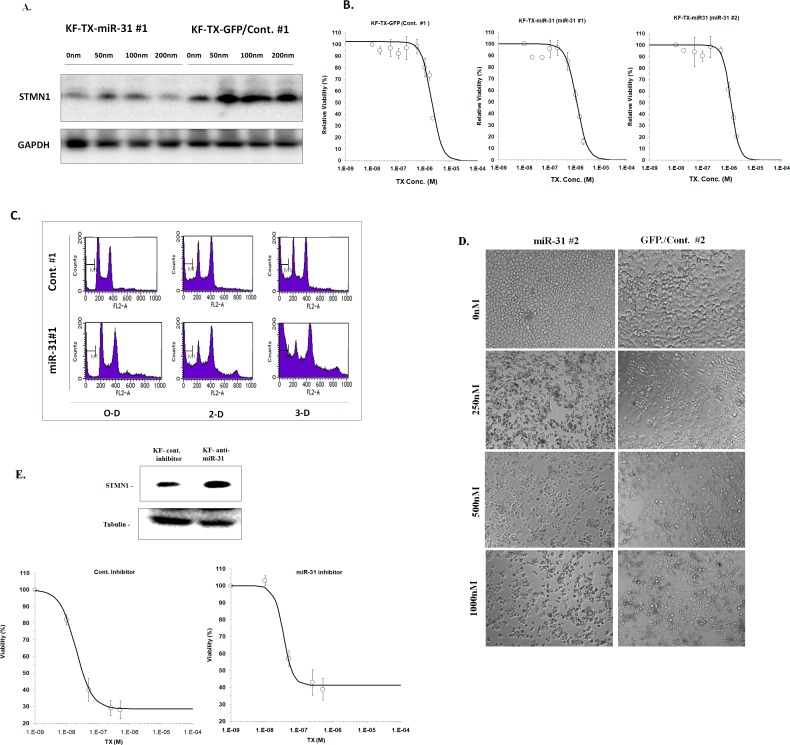
**A.** KF-TX cells show a differential expression profile of STMN1 in response to TX (different doses for 48 h) in miR-31 overexpressing cells compared to control. The KF-TX-miR-31 show less expression level of STMN1 gradually increase at 50nm treatment point and again decrease at 200nm. The Control (GFP) clone show relatively high expression of STMN1 which significantly increased and did not show significant reduction even at 200nm. B. miR-31 restores response to TX in KF-TX cells. Viability assay shows the differential response to TX in KF-TX-miR-31 (clones #1 and #2) and representative control (GFP only) clone (#1) for three days. The relative viability show that the clones overexpressing miR-31 are significantly sensitive to TX compared with the control. C. A representative FACS analysis shows the different apoptotic outcome of KF-TX cells clones overexpressing miR-31 (#1) and representative control clone (#1) after treatment with 250nM TX for the indicated time. D. Phase contrast of two different clones; control #2 and miR-31 #2 after 3-days of TX treatment at different doses for three days. E. Effect of miR-31 inhibitor on STMN1 expression. Immunobloting analysis of STMN1 in the parental cells, KF, transfected with the anti-miR-31 shows the relative upregulation of STMN1 compared with the control inhibitor (upper panel). Effect of anti-miR-31 on chemosensitivity to TX analyzed by viability assay in the parental KF cells. Anti-miR-31 confers chemoresistance in KF cells.

To study whether miR-31 could affect the STMN1 expression and was subsequently involved in chemoresponse, we established the stable clones overexpressing precursor miR-31 (KF-TX-miR31 #1 and #2). Microscopic observation for the expression of green florescent protein (Supplementary data; S.3) and quantitative RT-PCR for the amount of miR-31 (Figure 4A) confirmed overexpression of miR-31 in the stable clones, compared with control clones (KF-TX-control #1 and #2). miR-31 overexpression associated with reduced STMN1 expression as indicated by western blotting (Figure 4B) while a representative RT-PCR measurement confirmed the STMN1 transcripts' downregulation in the miR-31 overexpressing clone (clone #1; Figure 4B; right panel. In addition, TX-induced, dose-dependent, expression of STMN1 was significantly altered in miR-31-expressiong cells, which displayed a similar fashion, as observed in parental KF cells (Figure 5A, see also supplementary data; S.1). To examine whether the resistance to TX could be attenuated by miR-31expression in KF-TX cells, control and miR-31 overexpressing stable clones were treated with TX at various doses for three days. Viability assay shows the ability of miR-31 to resensitize KF-TX cells to TX (Figure 5B and supplementary data; S.4). The time dependent TX treatment and FACS analysis confirmed the enhanced response of KF-TX-miR-31 to TX as indicated by the sub G0 population (Figure 5C). Moreover morphological observation and phase contrast microscopic shots revealed that KF-TX-miR-31 cells are more sensitive to the same concentration of TX (200 nM, 500nM and 1000nM) compared with KF-TX-GFP control cells (Figure 5D).

Taken together, these data indicate that loss of miR-31 expression may play an important role in the development of TX-resistance in ovarian cancer cells, probably by allowing the upregulation of STMN1.

### Inhibition of miR-31 in KF cells confers TX resistance

To confirm that miR-31 mediates chemoresponse via modulation of STMN1 expression, we performed a reverse experiment, in which we transfected the parental KF cells with anti-miR-31 oligonucleotide (miR-31 inhibitor). Expectedly, the STMN1 expression had been enhanced by anti-miR-31 oligonucleotide transfection compared with a control oligonucleotide transfectant (Figure 5E; upper panel). Moreover, the viability test revealed that miR-31 inhibitor transfection confers TX resistance in the parental KF cells (Figure 5E; lower panels and supplementary data; S.5)

### STMN1 is depleted in the miR-31-overexpressing clones

**Figure 6:**
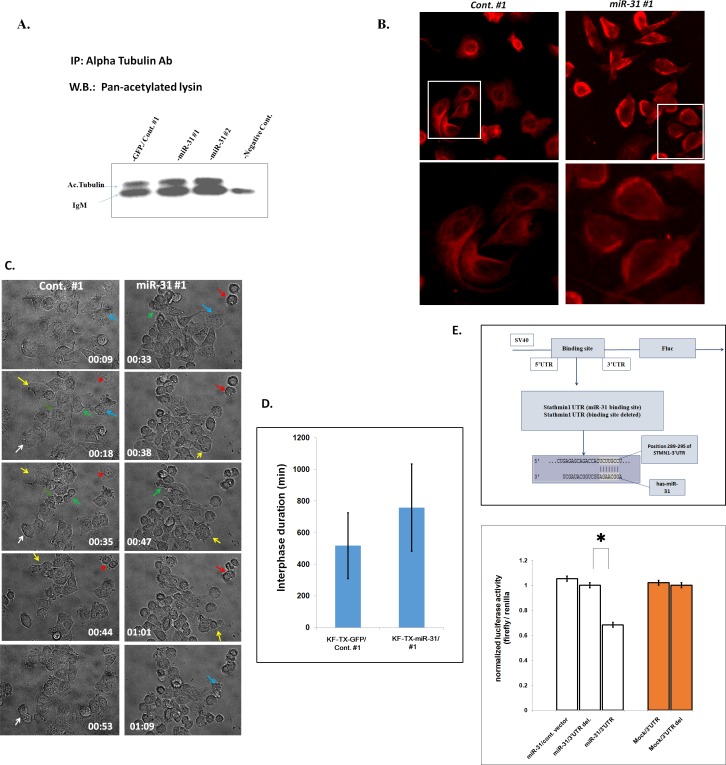
MiR-31 overexpressing KF-TX cells show increased acetylated α-tubulin levels A. Acetylated α-tubulin in the far immunoblot of α-tubulin immunoprecipitant from cell lysates isolated from either control/GFP or two stable clones overexpressing miR-31(#1 and #2). Acetylated tubulin is greater in KF-TX cells which overexpress miR-31, with depleted STMN1, compared with control transfectant which show higher STMN1. B. Immunocytochemical staining shows tubulin in KF-TX cells overexpressing miR-31 and their control (GFP). Representative images of a miR-31 clone (#1) and a control clone (#1) were fixed and stained with antibodies against α-tubulin. miR-31 overexpressing cells show more polymerized and bundled tubulin nearby the margins of the cells compared with normal tubulin distribution in the control as an evidence for STMN1 depletion in miR-31 clone compared with control one. miR-31 overexpression slows interphase in KF-TX cells. C. Phase contrast image obtained from time laps microscopic study (additional files; 1, 2). The series for a cell exiting mitosis. Plot summarizes data from >10 cells and three independent experiments per condition. STMN1 depletion increased interphase duration by approximately 210 min (**, P <0.05), compared with cells progressed through mitosis with normal kinetics. D. Immunoflorcent images show the distribution of the tubulin in the representative clones (KF-TX-miR-31; clone#1 and control-GFP #1). The miR-31 overexpressing cells showed bundled tubulin nearby the margimes of the cytoplasm E. Luciferase activity after transfection of the indicated 3′-UTR-driven reporter constructs. Reporter plasmid containing 3′-UTR region of STMN1 with binding site of miR-31 or with the binding site deleted, *P< 0.05.

STMN1 depletion leads to a G2 cell cycle delay and MT stabilization [43]. To verify whether miR-31 functionally modulates STMN1, we evaluated the MT stability by measuring the levels of acetylated α-tubulin. Western blotting analysis of the α-tubulin immunoprecipitant from stable clones using anti-acetylated lysine antibody indicates that miR-31 overexpressing cells contain higher acetylated tubulin compared with a control transfectant (Figure 6A). This result indicates that KF-TX-miR-31 contain more stable MTs, giving a mechanistic insight into the cellular consequences of STMN1 depletion. We also investigated the extent of microtubules polymerization in miR-31-expressing cells by confocal microscopy. Interestingly, KF-TX-miR-31 cells show different spatial pattern of microtubules distribution, the bundled microtubules nearby the cell periphery, compared with that in the control clone (Figure 6B).

STMN1 depletion was also reported to elongate interphase without affecting the mitotic duration [28,43]. We thus performed long-term live cell imaging to follow individual KF-TX-miR31 cells through cell cycles. A series of phase contrast images were acquired at 2-min intervals for 36 h and movies are developed (Additional files 1, 2; movies) for this purpose. Interphase was considered as the duration except for mitosis, which was also considered to begin with nuclear envelope disappearance and rounded shell shape, and terminate with formation of contraction ring. Consistent with our aforementioned results, as shown in figure 6C, miR-31 overexpression significantly slowed interphase duration compared with control cells, increasing the average interphase duration by 48% (Figure 6D). This increase is a typical marker for STMN1 depletion.

### MiR-31 directly down-regulates STMN1 expression

To elucidate rather, whether STMN1 is a direct target for miR-31, analysis using TargetScan database was performed to identify the interaction sequence from STMN1 transcript variant. The predicted binding of miR-31 with STMN1 3′UTR was illustrated (Figure 6E), indicating that miR-31 could potentially target STMN1. To further confirm that STMN1 is the direct target of miR-31, a firefly luciferase reporter vector containing a segment of the 3′UTR of STMN1 or the 3′UTR lacking the binding site (3′UTR del), was transfected into KF-TX control or KF-TX-miR-31 cells. Normalized luciferase activity in KF-TX-miR-31 cells transfected with the vector harboring STMN1 3′UTR was significantly lower than those in cells transfected with a control empty vector (luciferase cds only) or the vector harboring the binding site-deleted 3′UTR (Figure 6E). Luciferase activity in the control KF-TX cells was not altered irrespective of the presence or absence of the binding site. These results indicate that miR-31 directly suppressed STMN1 expression via sequence-specific interactions with 3′UTR of STMN1.

## DISCUSSION

Drug resistance is considered as a multifactorial phenomenon, involving several major mechanisms, such as decreased uptake of water-soluble drugs, increased repair of DNA damage, inhibition of apoptosis, altered metabolism of drugs, and increased energy-dependent efflux of chemotherapeutic drugs that diminishes the ability of cytotoxic agents to kill cancer cells. Although most of current studies are focused on cell apoptosis and drug transporters, a better understanding of other drug resistance mechanism may lead to the development of novel approaches for the treatment of ovarian and other cancers. Although TX mainly kills cancer cells by stabilizing MTs, stabilization of MTs by TX does not slow cell cycle progression through interphase, but rather blocks cells in mitosis [44, 45].

Recent findings have confirmed a critical role of miRNAs as powerful diagnostic and prognostic indicators of human ovarian cancer [31, 32,33]. Among miRNAs down-modulated in TX-resistant cells, we have previously reported, for the first time, that downregulation of miR-31 is associated with acquired resistance to TX [41]. The results of that study showed that overexpression of miR-31 in the KF-TX cells restored the sensitivity to TX, which provided a good rationale for the development of miRNA-based therapeutic strategies aiming to overcome cancer cell resistance to TX. This study focused on the role of STMN1 in ovarian cancer chemoresponse and added a novel target to miR-31, through which miR-31 can regulate ovarian cancer chemoresponse. Consistent with our result, overexpression of miR-31 inhibits ovarian cancer cell proliferation [42]. It is also reported that miR-31 inhibits cell proliferation, migration and invasion in malignant mesothelioma [46]. Furthermore, miR-31 was also reported to inhibit metastasis, whereas it enhances primary tumor growth of breast cancer [47]. Contributions of miR-31 to the activation of hypoxia-inducible factor for development of head and neck squamous cell carcinoma were also described [48]. Considering these reports, the roles of miR-31 in tumor cell proliferation and metastasis are complex. Creighton et al. described that miR-31 is downregulated in ovarian cancer tissues compared with normal ovarian surface epithelium, and is related to poor survival of ovarian cancer patients [42]. Give that poor survival is associated with ovarian cancer chemoresistence, together with our findings, we speculate that chemoresistance established by downregulation of miR-31 may explain the relation of miR-31 downregulation with poor survival of ovarian cancer, albeit in part. Alternatively, it may also possible that downregulation of miR-31 promotes metastatic potential of ovarian cancer cells, which might result in poor survival, as previously described in breast cancer cells [47].

One of the well-known functions of STMN1 is MT destabilization and reduced STMN1 level has an overall stabilizing effect on the MT cytoskeleton [49]. More specifically, STMN1 depletion increases the concentration of MT polymers and decreases the concentration of free tubulin dimers [49, 50]. In this study, we tried to clarify the mechanistic role of STMN1 in ovarian cancer TX-resistance development, and revealed that STMN1 expression was significantly increased in the chemoresistant ovarian cancer tissues compared to the responsive ones by immunochemistry. Furthermore, miRNA profiling indicated that miR-31 downregulation is responsible for STMN1 upregulation in chemoresistant ovarian cancer cells. Consistently, STMN1 overexpression has been associated with poor prognosis of ovarian cancer patients receiving TX-based chemotherapy [51]. Taken together, we thus speculate that chemoresistance established by downregulation of miR-31 could be explained, at least in part, by STMN1 upregulation.

In the current study, we first demonstrated that miR-31 directly represses expression of STMN1, induces microtubule polymerization, where the microtubules were bundled nearby the boundary of the cell, and subsequently modulates the sensitivity of a cell towards TX. Consistent with our results, STMN1 has been reported to be related to TX sensitivity in ovarian cancer and breast cancer as well [26, 51, 52], while targeting STMN1 by RNA interference can sensitize STMN1-overexpressing breast cancer cells to TX [26]. Second, we mapped out the negative relationship between STMN1 expression and miR-31 expression in ovarian cancer tissues. Opposite to the high expression of STMN1, miR-31 negatively associated to chemoresistance in ovarian cancer tissues.

Giving STMN1 a role in regulating the ovarian cancer chemoresponse, our data introduce miR-31 downregulation as the reason for STMN1 upregulation in response to TX treatment and resistance development. On the other side, introducing miR-31 to TX-resistant ovarian cancer cells not only reduced STMN1 expression followed by restoring chemoresponse, but also gave many signs that indicate the functional absence of STMN1, including MT stability and promotion of cell cycle progression from G2 to M phase. Therefore, STMN1 serves as a target for antimicrotubule therapies in ovarian cancer, consistent with the previous results shown in breast cancer [26]. In support of our finding, depleting STMN1 in many cancer cell lines slows cell proliferation due to a delay in cell cycle progression during the G2 phase and ultimately leads to apoptosis [24, 26, 28]. In contrast to cancer-derived cell lines, STMN1 depletion is not deleterious to nontransformed cells [25, 28]; in fact, STMN1 knockout mice are viable [53].

Importantly, it has been previously found that overexpression of STMN1 markedly affects the response to antimicrotubule agents including TX through at least two possible mechanisms: promoting microtubule destabilization and delaying the entry into M phase [52]. These mechanisms together lead to STMN1-mediated resistance to TX. Thus, up-regulation of STMN1 might be one of the mechanisms of resistance to TX regulated by downregulation of miR-31 in ovarian cancer cells.

In summary, the present study not only established that miR-31 is involved in the mechanism of TX-resistance in ovarian cancer cells, but also elucidated the biological function of miR-31, suggesting that the therapeutic delivery of miR-31 might restore TX sensitivity in ovarian cancer cells, as reported in breast cancer [47]. Finding other target genes that could be directly regulated by miR-31, and elucidating their role in chemoresistance to TX would open a window for further development of therapeutic approaches for TX-resistant ovarian cancer.

## MATERIALS AND METHODS

### Cell culture

The human serous ovarian cancer cell line SKOV3 was purchased from ATCC. The human serous ovarian cancer cell line KF and TU-OM-1 were kindly provided by Prof. Yoshihiro Kikuchi (National Defense Medical University) and Dr Hiroaki Itamochi (Tottori University School of Medicine), respectively. Cells were cultured in RPMI1640 medium supplemented with 10% fetal bovine serum (FBS) and 2 mM L-glutamine at 37°C in a 5% CO_2_ atmosphere. TX-resistant KF-TX subline was established from parental cell line KF by maintaining cells in increasing sublethal concentration of TX (starting from 10 nM for KF for more than ten months then IC_50_ of each clone was determined by the viability assay after three days treatment).

### Antibodies and reagents

Rabbit anti-human stathmin1 (STMN1) polyclonal antibody (Cell Signaling Technology, #3352, Danvers, MA, USA) was used for immunohistochemistry. Rabbit anti-human STMN1 polyclonal antibody (abcam, UK; # ab24445) and mouse anti-human α-tubulin monoclonal antibody (Sigma, USA) and mouse anti-pan acetylated lysine (Upstate Biotechnology, Lake Placid, NY, USA) were used for western blotting analysis. TX was supplied by Bristol-Myers Squib Co. Ltd. (Japan). We then prepared stock solution by diluting TX in the media at a final concentration of 4 μM and further working dilutions were carried out to reach the desired concentration.

### Human ovarian tumors

Tumor specimens from patients with ovarian cancer were obtained from Hokkaido University Hospital under institutional review board-approval. Informed consent was obtained from each patient. Patients treated at Hokkaido University Hospital between 1999 and 2012 were eligible. All samples were obtained at the initial surgery. miRNA was extracted by Recover-AllTM Total nucleic Acid Isolation Kit (Ambion) from formalin-fixed, paraffin-embedded tissues, of which epithelial tumors were confirmed by microscopic examination, and miR-31 was detected by quantitative real time PCR described below.

### Immunohistochemistry

Twenty-four formalin-fixed, paraffin-embed ovarian cancer tumor tissues were used for STMN1 expression investigation. Tissues were deparaffinized in xylene and rehydrated in descending ethanol series. Antigen retrieval was accomplished through microwave irradiation of the sections in 10 mM sodium citrate buffer. Slides were incubated with rabbit anti-STMN1 polyclonal antibody, and then incubated with biotin-conjugated goat anti-rabbit IgG (Jackson Immunoresearch Laboratories, West Grove, PA, USA). The bound immune complexes were developed by addition of diaminobenzidine (DAB; Sigma-Aldrich, St. Louis, MO, USA) and the nuclei were stained with hematoxylin (Sigma-Aldrich). The sections were also incubated with normal goat serum as anegative control. Samples were viewed using Nikon TE 2000-U microscope (NIKON, Tokyo, Japan). All of the slides were reviewed by three full-boarded pathologists without knowledge of the clinical data. Immunohistochemical positivities were evaluated by proportion and intensity. For analysis of proportion, four tired evaluation was applied as 0 to 3: no staining (0), 1–10% (1), 11–50% (2) and 51–100% of tumor cells (3). For evaluation of intensity, we used following four criteria: negative (0;-), weak (1;+), intermediate (2;++) and strong (3;+++). STMN1 immunohistochemistry score was shown as sum of proportional and intensity scores (0 to 6).

### RNA extraction and qRT-PCR

For quantification of pre-miR-31, TaqMan® miRNA assay was performed. Total RNAs containing miRNAs was extracted using miRNeasy Mini Kit (QIAGEN, Germany) and subjected to reverse transcription (RT) reaction using TaqMan® MicroRNA RT Kit (Applied Biosystems). Real time RT-PCR was performed using TaqMan® MicroRNA Assay kit (Applied Biosystems). TaqMan® microRNA assays miR-31 (cat#4373190) was used to quantify miR-31 expression. TaqMan® microRNA assays RNU44 (cat#4373384) and TaqMan® microRNA assays RNU48 (cat#4373383) were used to quantify RNU44 and RNU48 as internal controls, respectively. Expression level of miR-31 was normalized to RNU44 and RNU48. Data were analyzed by using ABI PRISM 7900HT sequence detection system (Applied Biosystems) and ABI PRISM SDS2.1 (Applied Biosystems).

For mRNA quantification, total RNA was extracted using TRIzol Reagent (Invitrogen), and RT was performed for 2 μg RNA using SuperScript II Reverse Transcriptase (Invitrogen) following the manufacturer's instruction. The PCR amplification was performed using GoTaq Green Master Mix (Promega). The mRNA level of stmn1 and glyceraldehyde-3-phosphate dehydrogenase (GAPDH) was amplified by a FastStart Universal SYBR Green Master (Roche, Mannheim, Germany) and StepOneTM Real-Time PCR System (Applied Biosystems, Foster City, CA, USA). The expression level was normalized to that of Gapdh. Primers used for expression analysis were as follows: GAPDH-fowward, 5′-CTCATGACCACAGTCCATGC-3′; GAPDH-reverse, 5′-TTACTCCTTGGAGGCCATGT-3′; STMN1-forward, 5′-TGGCAGAAGAGAAACTGACCCACA; STMN1-reverse, 5′-TCTCGTCAGCAGGGTCTTTGGATT-3′ [37]. Each sample in each group was measured in triplicate and the experiment was repeated at least three times.

### Establishment of stable clones overexpressing miR-31

pMIF-GFP/pre-miR-31-zeo vector expressing precursor for miR-31 was purchased from Funakoshi (cat#MIFCZ312PA-1, Japan). KF-TX cells were cultured to 50% confluence. Plasmid DNA transfection was done using Effectine (Qiagen, Germany) according to the manufacturer's instructions. KF-TX cells were either transfected with pMIF-GFP/pre-miR-31-zeo plasmid or co-transfected with the empty vector pZeo with pGFP and in ratio of 3:2. Then cells were selected in zeocin (100 μg/ml; Invitrogen) for three weeks. Stable expression of miR-31 was confirmed by GFP expression and quantitative RT-PCR described above.

### Cell viability determination and morphological evaluation

Cell viability was detected using cell counting kit (CCK-8) (Dojindo, Japan). Briefly, cells from different clones were pre-cultured in 96-well plate (3,000 cells/well) for 24 h. Seventy two hours after TX treatment at the indicated doses, culture media were replaced by the WST-8 reagent [2-(2-methoxy-4-nitrophenyl)-3-(4-nitrophenyl)-5-2,4disulphonyl]-2H-tetrazolium, monosodium salt]. WST-8 reduced by the cellular dehydrogenases turns into orange formazan. The amount of formazan produced is directly proportional to the number of living cells. Color developed and the absorbance at 450 nm was measured by a microplate reader (NEC, Tokyo, Japan). To morphologically evaluate cell death, cells were visualized under a phase-contrast microscope.

### Flow cytometry analysis

Following TX treatment, cells were trypsinized and washed twice in phosphate-buffered saline (PBS); cell cycle phases were then analyzed as described [38] with a minor modification. Briefly, cells were fixed at 4°C overnight in 70% ethanol. After washing with Ca^2+^-Mg^2+^-free Dulbecco's PBS, cells were treated with 0.1 μg/ml RNase (Type I-A, Sigma), stained with 100 ug/ml propidium iodide (PI; Sigma) for 20 min, filtered, and kept on ice until measurement. Cells were measured by the FACSCalibur^TM^ (Becton Dickinson) and the data obtained were then analyzed using ModFit software. Cell fractions with a DNA content lower than G0/G1, the sub-G0/G1 peak, were quantified and considered a marker of the number of apoptotic cells.

### Western blot analysis

Cells were lysed in lysis buffer [10 mmol/L Tris-HCl (pH 7.4), 150 mmol/L NaCl, 1 mmol/L EDTA, 0.5% NP40, 50 mmol/L NaF, 1 mmol/L phenylmethylsulfonyl fluoride, 1 mmol/L Na_3_ VO_4_]. Protein concentration of whole cell lysates was determined by BSA assay using the BSA kit (Pierce, Rockford, IL), and equal protein amounts were then boiled at 99°C for 5 min with SDS-polyacrylamide gel electrophoresis (PAGE) sample buffer (25% glycerol, 31.2 ml 0.25M Tris-HCl pH 6.8, 7.5 ml 10% SDS, 0.3M DTT and a dash of bromophenol blue/100ml) and separated by 14%, 12% SDS-PAGE for STMN1. Separated proteins were then transferred onto a polyvinylidene difluoride (PVDF) membranes (Millipore) following standard methods. The membranes were incubated in blocking solution (2% non-fat milk in PBS) for 1h, further incubated with primary antibodies (anti-human STMN1 at a dilution of 1:500 and anti-human tubulin at a dilution of 1:2000) overnight at 4oC, and then incubated with an secondary antibody (anti-rabbit antibody at a dilution of 1:1000 for STMN1 or anti-mouse antibody at a dilution of 1:1000 for tubulin) for 1 h at room temperature. After 3 × 10-min washes in T-PBS (0.1% Tween-20 in PBS) the membrane was incubated for 1 h at room temperature with horseradish peroxidase the following primers: Stathmin1 3′-UTR-forward, (HRP)-linked IgG as secondary antibody (1:5,000 dilution in T-TBS) followed by three washes (10 min each) with T-TBS. Signal on membranes was developed using ECL reagent (Amersham, USA) and then was imaged by Lumino Image Analyzer (LAS1000, Fuji Film, Tokyo, Japan).

### miRNA inhibitor

Two hundred and fifty nanomolar of Anti-hsa-miR-3, miScript miRNA Inhibitor, targets mature miRNA-31 and its negative control (Qiagen, Germenay) were employed to transiently inhibit miR-31. Cells were transfected 48 h with HiPerfect before seeding for further experiment (Qiagen)

### Dual-luciferase Activity Assay

To generate 3′-UTR luciferase reporter, partial sequence of the 3′-UTR from stmn1 were cloned into the downstream of the firefly luciferase gene in pGL4.11 control vector (Promega, Madison, WI, USA). The 3′-UTR of stmn1 with the miR-31 target site deleted was used as a control. pRL-TK containing Renilla luciferase was cotransfected for data normalization. For luciferase reporter assays, KF-TX cells were seeded in 48-well plates, allowed to attach overnight, and then transfected using Lipofectamine 2000 (Invitrogen, Carlsbad, CA, USA). Two days later, cells were harvested and assayed with the dual-luciferase assay (Promega, Madison, WI, USA). Each treatment was performed in triplicate in three independent experiments. The results were expressed as relative luciferase activity (Firefly LUC/Renilla LUC).

The Stmn1 3′-UTR and 3-UTR-mutant sequences were amplified by PCR from human genomic DNA using 5′-GGACTAGTGAACTGACTTTCTCCCCATCCC-3′; STMN1 3′-UTR-reverse, 5′-AACTGCAGGCCACCAACAGCACTGTG-3′; Stmn1 3′-UTR (deleting binding region) forward: 5′-GGACTAGTGAACTGACTTTCTCCCCATCCC-3′; reverse, 5′-TAGCCATTAACCCAGTACACCAAG-3′[37]. The PCR products were cloned into pCR-BluntII-TOPO vector using the Zero Blunt® TOPO® PCR Cloning Kit (Invitrogen). The desired DNA was then restricted by *Sac*I and *Xho*I, and subcloned into the *Sac*I and *Xho*I sites of the pGL4.11 vector.

### Live cell imaging

To follow cell fates over several hours, cells were plated on Mattek dishes and were imaged using a Nikon Biostation IM (Nikon, Melville, NY). Throughout imaging, cells were maintained in a humidified chamber and in a 5% CO_2_ atmosphere provided by an MIGM Gas Mixer (Tokken, Japan). Cells were imaged with phase contrast optics using a 20× objective and images were collected at 2-min intervals for 36 h. Cell fates were tracked from the image series. Mitotic entry was scored either by the first image showing loss of nuclear envelope integrity or by extensive cell rounding that makes it impossible to see the nuclear envelope. Mitotic exit was scored as the first image showing indentation of the plasma membrane, indicative of the beginning of cytokinesis.

### Statistical analysis

Statistical analysis was performed using Minitab Release (Ver.12). Data were subjected to one-way analysis of variance, followed by comparison using student *t* test to evaluate the difference between means. Differences between means were considered significant if p-values <0.05.

## SUPPLEMENTARY MATERIAL FIGURES







## References

[1] Ahmed FY1, Wiltshaw E, A'Hern RP, Nicol B, Shepherd J, Blake P, Fisher C, Gore ME (1996). Natural history and prognosis of untreated stage I epithelial ovarian carcinoma. J Clin Oncol.

[2] Garcia M, Jemal A, Ward EM, Center MM, Hao Y, Siegel R (2007). Global cancer facts and figures.

[3] Miller M, Ojima I (2001). Chemistry and chemical biology of taxane anticancer agents. Chem Rec.

[4] Petrylak D (2003). Docetaxel for the treatment of hormone-refractory prostate cancer. Rev Urol.

[5] Fung-Kee-Fung M, Oliver T, Elit L, Oza A, Hirte HW, Bryson P (2007). Optimal chemotherapy treatment for women with recurrent ovarian cancer. Curr Oncol.

[6] Bergstralh DT, Ting JP.Y (2006). Microtubule stabilizing agents: their molecular signalling consequences and the potential for enhancement by drug combination. Cancer Treat Rev.

[7] Xiao H1, Verdier-Pinard P, Fernandez-Fuentes N, Burd B, Angeletti R, Fiser A, Horwitz SB, Orr GA (2006). Insights into the mechanism of microtubule stabilization by Taxol. Proc Natl Acad Sci U S A.

[8] Horwitz SB, Shen HJ, He L, Dittmar P, Neef R, Chen J, Schubart UK (1997). The microtubule-destabilizing activity of metablastin (p19) is controlled by phosphorylation. J Biol Chem.

[9] Gavet O, Ozon S, Manceau V, Lawler S, Curmi P, Sobel A (1998). The stathmin phosphoprotein family: intracellular localization and effects on the microtubule network. J Cell Sci.

[10] Wallon G, Rappsilber J, Mann M, Serrano L (2000). Model for stathmin/OP18 binding to tubulin. EMBO J.

[11] Belmont LD1, Mitchison TJ (1996). Identification of a protein that interacts with tubulin dimers and increases the catastrophe rate of microtubules. Cell.

[12] Curmi PA, Gavet O, Charbaut E, Ozon S, Lachkar-Colmerauer S, Manceau V, Siavoshian S, Maucuer A, Sobel A (1999). Stathmin and its phosphoprotein family: general properties, biochemical and functional interaction with tubulin. Cell Struct Funct.

[13] Howell B, Deacon H, Cassimeris L (1999). Decreasing oncoprotein 18/stathmin levels reduces microtubule catastrophes and increases microtubule polymer in vivo. J Cell Sci.

[14] Martín L, Fanarraga ML, Aloria K, Zabala JC (2000). Tubulin folding cofactor D is a microtubule destabilizing protein. FEBS Lett.

[15] Cheon MS, Fountoulakis M, Cairns NJ, Dierssen M, Herkner K, Lubec G (2001). Decreased protein levels of stathmin in adult brains with Down syndrome and Alzheimer's disease. J Neural Transm Suppl.

[16] Liedtke W, Leman EE, Fyffe RE, Raine CS, Schubart UK (2002). Stathmin-deficient mice develop an age-dependent axonopathy of the central and peripheral nervous systems. Am J Pathol.

[17] Hailat N, Strahler J, Melhem R, Zhu XX, Brodeur G, Seeger RC, Reynolds CP, Hanash S (1990). N-myc gene amplification in neuroblastoma is associated with altered phosphorylation of a proliferation related polypeptide (Op18). Oncogene.

[18] Campostrini N, Pascali J, Hamdan M, Astner H, Marimpietri D, Pastorino F, Ponzoni M, Righetti PG (2004). Proteomic analysis of an orthotopic neuroblastoma xenograft animal model. J Chromatogr B Analyt Technol Biomed Life Sci.

[19] Don S, Verrills NM, Liaw TY, Liu ML, Norris MD, Haber M, Kavallaris M (2004). Neuronal-associated microtubule proteins class III beta-tubulin and MAP2c in neuroblastoma: role in resistance to microtubule-targeted drugs. Mol Cancer Ther.

[20] Brattsand G (2000). Correlation of oncoprotein 18/stathmin expression in human breast cancer with established prognostic factors. Br J Cancer.

[21] Curmi PA, Noguès C, Lachkar S, Carelle N, Gonthier MP, Sobel A, Lidereau R, Bièche I (2000). Overexpression of stathmin in breast carcinomas points out to highly proliferative tumours. Br J Cancer.

[22] Johnsson A, Zeelenberg I, Min Y, Hilinski J, Berry C, Howell SB, Los G (2000). Identification of genes differentially expressed in association with acquired cisplatin resistance. Br J Cancer.

[23] Price DK1, Ball JR, Bahrani-Mostafavi Z, Vachris JC, Kaufman JS, Naumann RW, Higgins RV, Hall JB (2000). The phosphoprotein Op/stathmin is differentially expressed in ovarian cancer. Cancer Invest.

[24] Mistry SJ, Bank A, Atweh GF (2005). Targeting stathmin in prostate cancer. Mol Cancer Ther.

[25] Zhang D, Tari AM, Akar U, Arun BK, LaFortune TA, Nieves-Alicea R, Hortobagyi GN, Ueno NT (2010). Silencing kinase-interacting stathmin gene enhances erlotinib sensitivity by inhibiting Ser^10^ p27 phosphorylation in epidermal growth factor receptor-expressing breast cancer. Mol Cancer Ther.

[26] Alli E, Yang JM, Hait WN (2007). Silencing of stathmin induces tumor-suppressor function in breast cancer cell lines harboring mutant p53. Oncogene.

[27] Wang R, Dong K, Lin F, Wang X, Gao P, Wei SH, Cheng SY, Zhang HZ (2007). Inhibiting proliferation and enhancing chemosensitivity to taxanes in osteosarcoma cells by RNA interference-mediated downregulation of stathmin expression. Mol Med.

[28] Carney BK, Cassimeris L (2010). Stathmin/oncoprotein 18, a microtubule regulatory protein, is required for survival of both normal and cancer cell lines lacking the tumor suppressor, p53. Cancer Biol Ther.

[29] Wu L, Belasco J. (2008). Let me count the ways: mechanisms of gene regulation by miRNAs and siRNAs. Mol Cell.

[30] Slack F, Weidhaas J (2008). MicroRNA in cancer prognosis. N Engl J Med.

[31] Dahiya N, Sherman-Baust C, Wang T, Davidson B, Shin Ie-M, Zhang Y, Wood W (2008). MicroRNA expression and identification of putative miRNA targets in ovarian cancer. PLoS One.

[32] Nam E, Yoon H, Kim S, Kim H, Kim Y, Kim J, Kim J, Kim S (2008). Clin Cancer Res.

[33] Iorio M, Visone R, Di Leva G, Donati V, Petrocca F, Casalini P, Taccioli C, Volinia S, Liu C, Alder H, Calin G, Menard S, Croce C (2007). MicroRNA signatures in human ovarian cancer. Cancer Res.

[34] Zhang L, Volinia S, Bonome T, Calin G, Greshock J, Yang N, Liu C, Giannakakis A, Alexiou P, Hasegawa K, Johnstone C, Megraw M, Adams S, Lassus H, Huang J, Kaur S, Liang S, Sethupathy P, Leminen A, imossis V, Sandaltzopoulos R, Naomoto Y, Katsaros D, Gimotty P, DeMichele A, Huang Q, Bützow R, Rustgi A, Weber B, Birrer M, Hatzigeorgiou A, Croce C, Coukos G (2008). Genomic and epigenetic alterations deregulate microRNA expression in human epithelial ovarian cancer. Proc Nat Acad Sci U S A.

[35] Tricoli J, Jacobson J (2007). MicroRNA potential for cancer detection, diagnosis, and prognosis. Cancer Res.

[36] Zheng T, Wang J, Chen X, Liu L (2010). Role of microRNA in anticancer drug resistance. Int J Cancer.

[37] Wang R, Wang HB, Hao CJ, Cui Y, Han XC, Hu Y, Li FF, Xia HF, Ma X (2012). MiR-101 is involved in human breast carcinogenesis by targeting Stathmin1. PLoS One.

[38] Nicoletti I, Migliorati G, Pagliacci MC, Grignani F, Riccardi C (1991). A rapid and simple method for measuring thymocyte apoptosis by propidium iodide staining and flow cytometry. J Immunol Methods.

[39] Benedet JL, Bender H, Jones H, Ngan HY, Pecorelli S (2000). FIGO staging classifications and clinical practice guidelines in the management of gynecologic cancers. FIGO. Committee on Gynecologic Oncology. Int J Gynaecol Obstet.

[40] Eisenhauer EA, Therasse P, Bogaerts J, Schwartz LH, Sargent D, Ford R (2009). New. response evaluation criteria in solid tumours: revised RECIST guideline. Eur J Cancer.

[41] Mitamura T, Watari H, Wang L, Kanno H, Hassan MK, Miyazaki M, Katoh Y, Kimura T, Tanino M, Nishihara H, Tanaka S, Sakuragi N (2013). Downregulation of miRNA-31 induces taxane resistance in ovarian cancer cells through increase of receptor tyrosine kinase MET. Oncogenesis.

[42] Creighton CJ, Fountain MD, Yu Z, Nagaraja AK, Zhu H, Khan M (2010). Molecular profiling uncovers a p53-associated role for microRNA-31 in inhibiting the proliferation of serous ovarian carcinomas and other cancers. Cancer Res.

[43] Carney BK, Caruso Silva V, Cassimeris L (2012). The microtubule cytoskeleton is required for a G2 cell cycle delay in cancer cells lacking stathmin and p53. Cytoskeleton (Hoboken).

[44] Horwitz SB (1992). Mechanism of action of taxol. Trends Pharmacol Sci.

[45] Uetake Y, Sluder G (2007). Cell-cycle progression without an intact microtuble cytoskeleton. Curr Biol.

[46] Ivanov SV, Goparaju CM, Lopez P, Zavadil J, Toren-Haritan G, Rosenwald S, Hoshen M, Chajut A, Cohen D, Pass HI (2010). Pro-tumorigenic effects of miR-31 loss in mesothelioma. J Biol Chem.

[47] Valastyan S, Reinhardt F, Benaich N, Calogrias D, Szász AM, Wang ZC, Brock JE, Richardson AL, Weinberg RA (2009). A pleiotropically acting microRNA, miR-31, inhibits breast cancer metastasis. Cell.

[48] Liu CJ, Tsai MM, Hung PS, Kao SY, Liu TY, Wu KJ, Chiou SH, Lin SC, Chang KW (2010). miR-31 ablates expression of the HIF regulatory factor FIH to activate the HIF pathway in head and neck carcinoma. Cancer Res.

[49] Sellin ME, Holmfeldt P, Stenmark S, Gullberg M (2008). Op18/Stathmin counteracts the activity of overexpressed tubulin-disrupting proteins in a human leukemia cell line. Exp Cell Res.

[50] Ringhoff DN1, Cassimeris L (2009). Stathmin regulates centrosomal nucleation of microtubules and tubulin dimer/polymer partitioning. Mol Biol Cell.

[51] Su D, Smith SM, Preti M, Schwartz P, Rutherford TJ, Menato G, Danese S, Ma S, Yu H, Katsaros D (2009). Stathmin and tubulin expression and survival of ovarian cancer patients receiving platinum treatment with and without paclitaxel. Cancer.

[52] Alli E1, Bash-Babula J, Yang JM, Hait WN (2002). Effect of stathmin on the sensitivity to antimicrotubule drugs in human breast cancer. Cancer Res.

[53] Schubart UK, Yu J, Amat JA, Wang Z, Hoffmann MK, Edelmann W (1996). Normal development of mice lacking metablastin (P19), a phosphoprotein implicated in cell cycle regulation. J Biol Chem.

