# Supplementary material for: P18/Stathmin1 is regulated by miR-31 in ovarian cancer in response to taxane
Source: Oncoscience. 2015 Mar 23;2(3):294–308. doi: 10.18632/oncoscience.143 (PMC4394135; doi:10.18632/oncoscience.143)
Supplement: Supplementary file 1 [file oncoscience-02-0294-s001.pdf]

P18/Stathmin1 is regulated by miR-31 in ovarian cancer in response to taxane

Supplementary Material

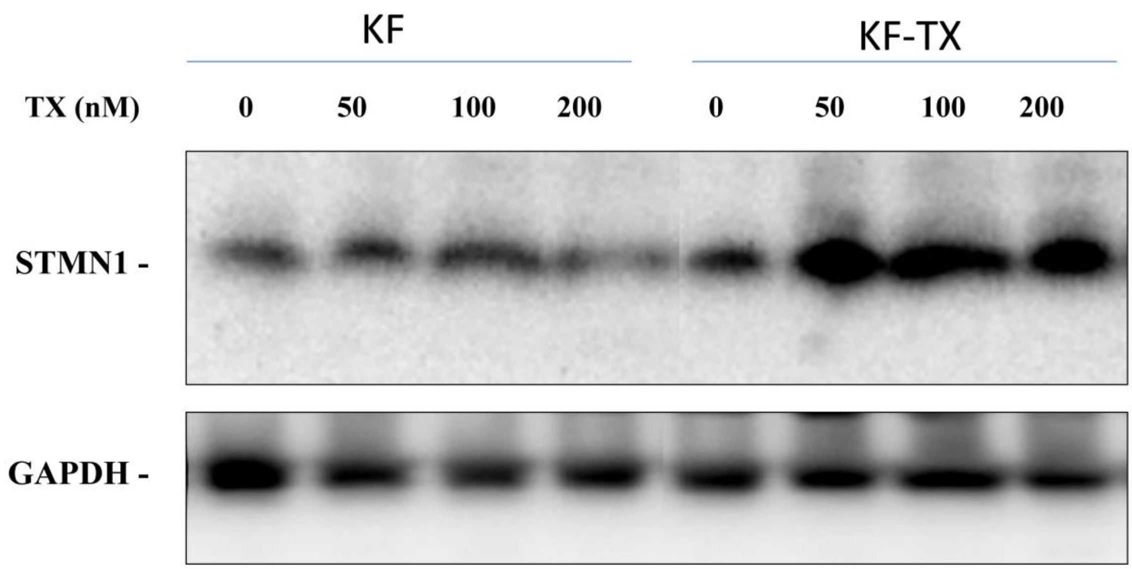

S.1. STMN1 is upregulated in KF-TX cells. Immunnoblting analysis of STMN1 in KF and KF-TX after treatment with different doses (0nM-200nM) of TX. The KF-TX cells show enhanced STMN1 expression in response to TX compared with, parental, KF cells.

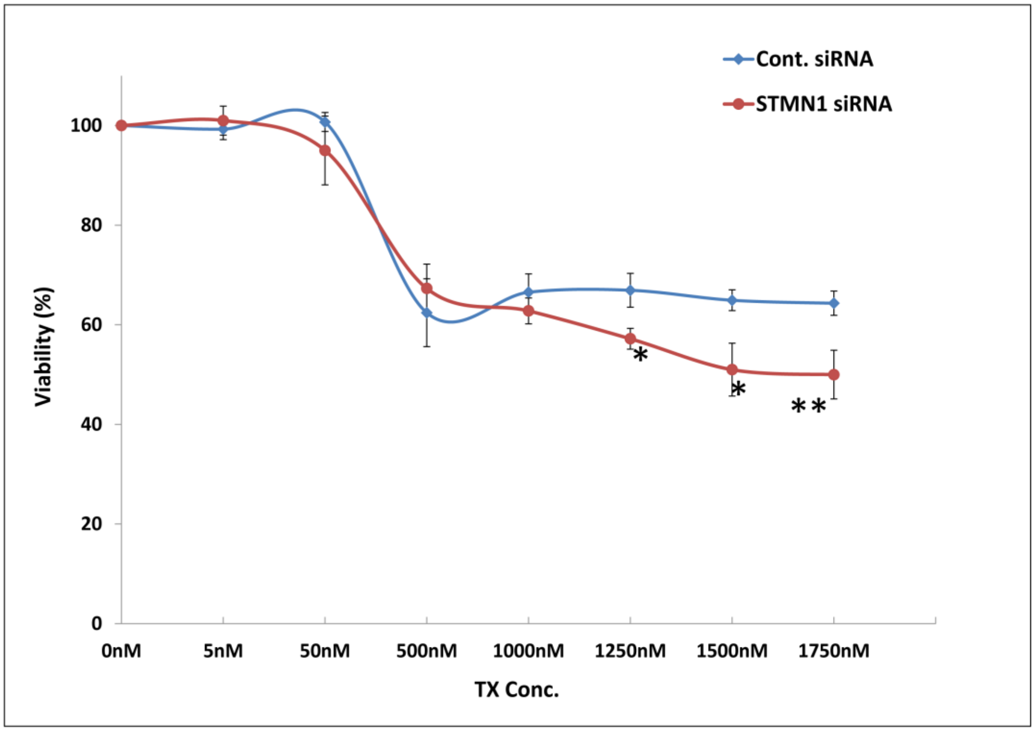

S.2 STMN1 knock down enhanced apoptotic cell death by TX in KF-TX cells. KF-TX cells were transfected with STMN1 siRNA or control siRNA (100 nM) twice. One day after last transfection, equal cell numbers were subcultured for further 24h, and then treated with TX for three days. Each data point represents mean of three experiments; bars denote SDV; * indicate difference from control transfectant at P≤ 0.001**

A

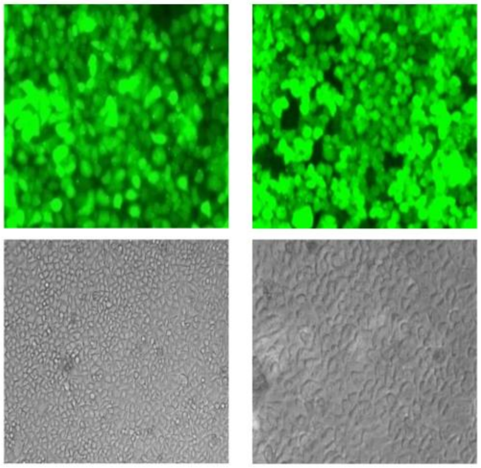

Cont./GFP only miR-31 Clone #1

B

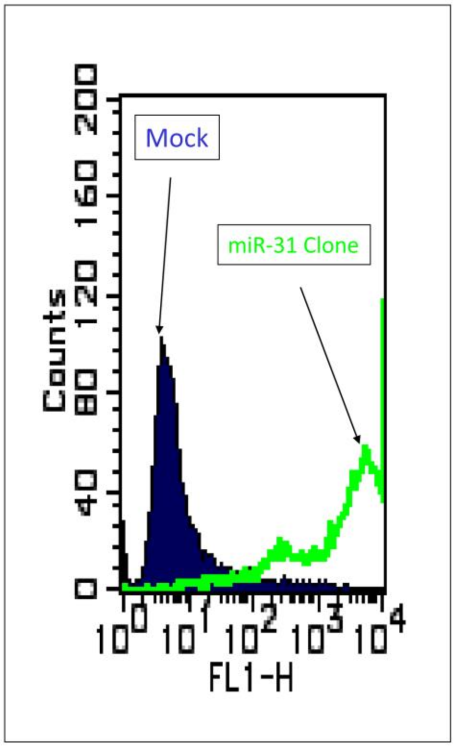

S.3 Validation of stable clone (KF-TX-miR-31) establishment. A. A flourscent images showing the GFP in both control (GFP only) and mir-31 expressing vector-transfected cells in KF-TX cells. B. FACS analysis showing the shift of the majority of cell population in the stable clone indicating the expression of GFP in almost all cells in a representative clone.

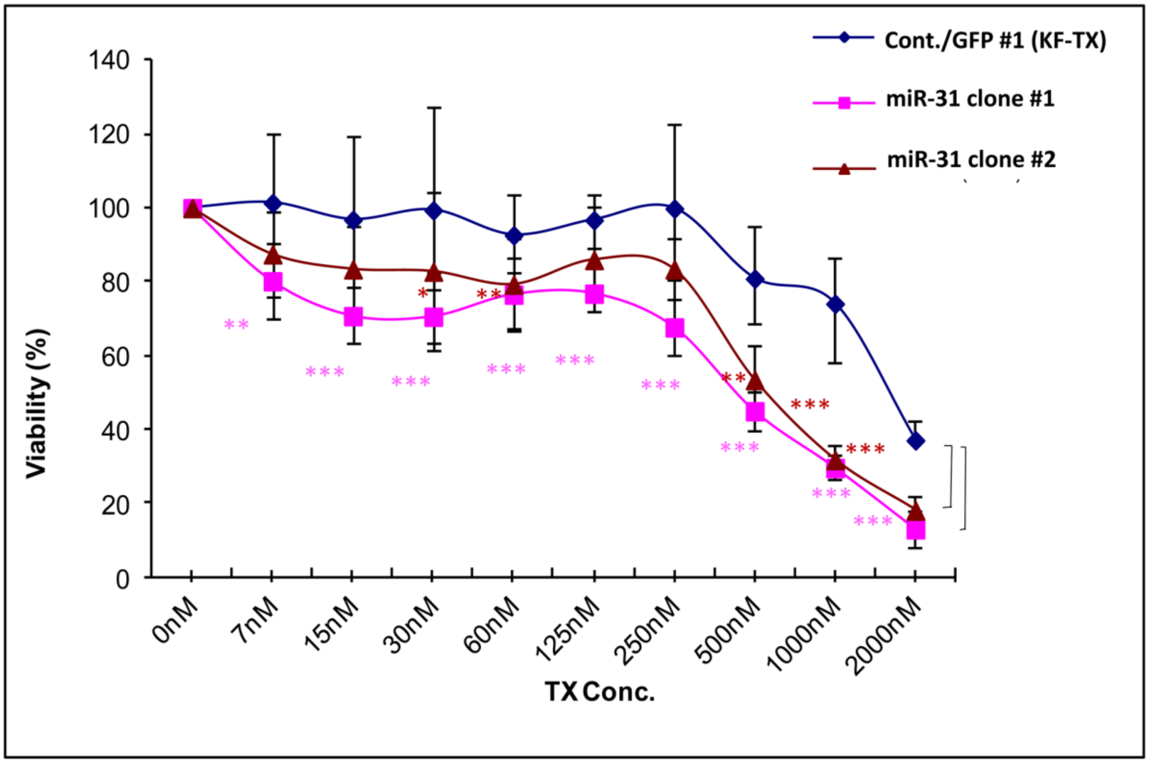

S. 4 miR-31 expression restores response to TX in KF-TX cells. Viability assay shows the differential response to TX in KF-TX-miR-31 (clones #1 and #2) and representative control (GFP only),clone #1, for three days. The relative viability show that the clones overexpressing miR-31 are significantly sensitive to TX compared with the control.

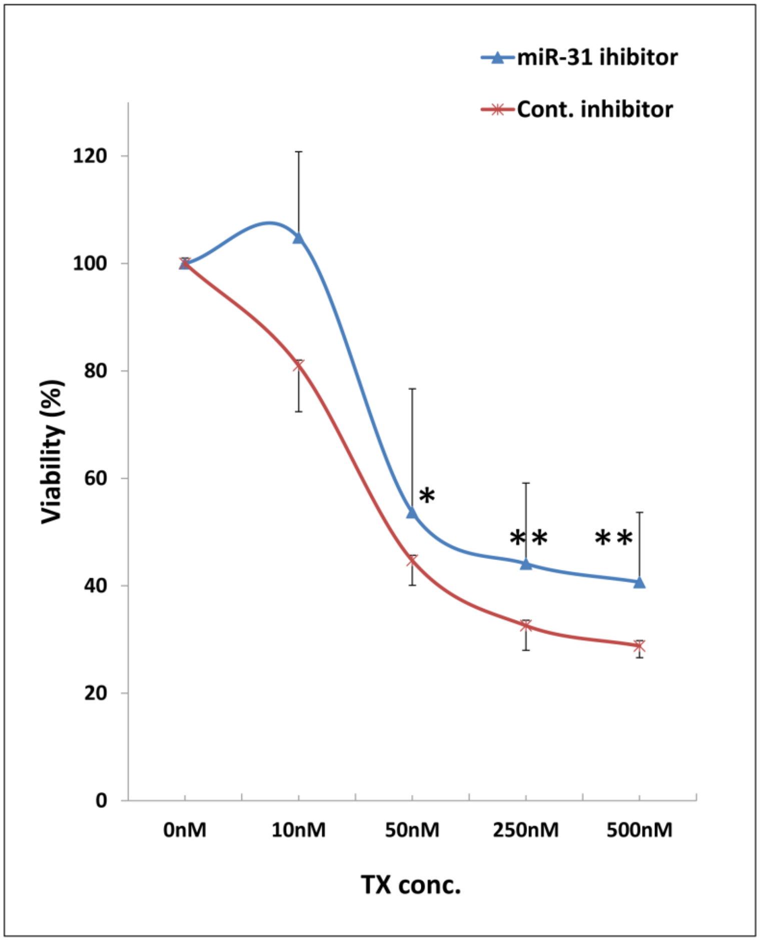

S.5 Anti-miR-31 confers chemoresistance in KF cells. Effect of anti-miR-31 on the response to TX in the parental KF cells. Cellular viability was estimated in the parental KF cells, * denotes P<0.05.
